# Exploring the impact of SNPs rs2476601, rs2488457, and rs33996649 on *PTPN22* expression, structure, and anti-CCP level in rheumatoid arthritis of the Indian population: a case-control and computational study

**DOI:** 10.3389/fmed.2025.1688505

**Published:** 2025-10-21

**Authors:** S. Aswini, S. Asha Devi

**Affiliations:** Department of Biomedical Sciences, School of Biosciences and Technology, Vellore Institute of Technology, Vellore, India

**Keywords:** rheumatoid arthritis, rs2476601, rs2488457, genotyping, MDS, HRMA, anti-CCP antibody

## Abstract

**Background:**

Protein Tyrosine Phosphatase Non-Receptor Type 22 (*PTPN22*) gene encodes for Lymphoid-Specific Tyrosine Phosphatase (LYP), predominantly expressed in T lymphocytes to regulate the immune responses by modulating T-cell proliferation and activation. Single Nucleotide Polymorphisms (SNPs) in *PTPN22* alter the structure and function of LYP, leading to dysregulation of T-cell response and increasing the risk for autoimmune disease.

**Methods:**

To investigate the intricate relationships between *PTPN22* SNPs rs2476601, rs2488457, and rs33996649 and Rheumatoid Arthritis (RA) pathogenesis, genotyping was accomplished using High-Resolution Melting Analysis (HRMA) and Sanger sequencing in 229 RA and 239 control samples. The SNP rs2476601 disrupts the interaction between PTPN22 and Peptidyl arginine deiminase, which leads to an altered production of citrullinated proteins and consequently raises anti-CCP autoantibody levels. Hence, we further analyzed the impact of SNP rs2476601 on serum anti-CCP antibody levels in RA patients, and structural difference between the PTPN22 wild and R620W variant proteins by Molecular Dynamics Simulation (MDS). We also investigated the *PTPN22* rs2488457 variant’s effect on *PTPN22* mRNA expression level, noting its location in the gene’s promoter region, which may affect transcription and expression level. The influence of rs2488457 on *PTPN22* mRNA expression level was determined by comparative threshold cycle method.

**Results:**

The statistical analysis confirms that RA samples with SNP rs2476601 C/T genotype (Odds Ratio (OR) = 3.6136; Confidence Interval (CI) = 2.2789 to 5.7300; *p* = 0.0001) and rs2488457 C/C genotype (OR = 1.5179; CI = 1.0453 to 2.2042; *p* = 0.0283) were associated with RA. SNP rs2476601 C/T genotype also elevated the serum anti-CCP antibody levels significantly (*p* = 0.01). The MDS result revealed significant structural and dynamic differences between wild and R620W variant forms of the PTPN22 protein. Furthermore, individuals with RA carrying the C/C genotype at the rs2488457 locus significantly (*p* = 0.0350) downregulated the *PTPN22* mRNA expression level.

**Conclusion:**

This study suggests that *PTPN22* SNPs rs2476601, rs2488457 are strongly linked with RA susceptibility in the Indian ethnicity.

## 1 Introduction

Rheumatoid Arthritis (RA) is a long-lasting, crippling inflammatory condition that affects synovial tissues, particularly in the extremities (1). Compared to other autoimmune illnesses, the origin of RA is not precisely understood; however, studies on twins, families, and genome-wide linkage scans (2–4) have reported that the heritability of RA is approximately 50–60%, indicating that genetic element has a significant influence on RA. The primary hallmarks of autoimmune diseases are immune system dysregulation, which results in self-reactive T-cell development. As a result, controlling T-cell activation and proliferation through co-inhibitory molecules aids in the treatment of various autoimmune disorders. Protein Tyrosine Phosphatase Non-Receptor Type 22 (*PTPN22*) gene, positioned on the chromosome 1p13.3–13.1, encodes for a non-receptor protein with 807 amino acids, known as Lymphoid-Specific Tyrosine Phosphatase (LYP) (5) or Protein Tyrosine Phosphatase. LYP is predominantly expressed in T-lymphocytes and acts as a negative regulator to suppress the differentiation and activation of T cells. The protein has three critical domains, including the N-terminal catalytic or PTP domain (1–300 amino acids), an interdomain region (301–600 amino acids), and four proline-rich C-terminal domains (601–807 amino acids). T-cell activation via the T-cell receptor (TCR) depends on the sequential phosphorylation of tyrosine residues mediated by kinases of the Src and Syk families. In the T-cell signaling pathway, the proline-rich motif of LYP binds with the SH3 domain of C-terminal Src Kinase (CSK) to form a stoichiometric complex. The LYP-CSK complex dephosphorylates tyrosine residues of intracellular proteins such as Lck, Fyn (Src kinase), ZAP-70 (Syk kinase), and immunoreceptor tyrosine-based activation motif of fragment crystallizable receptor-like molecule protein, which are involved in the TCR downstream signaling pathway to inhibit the T-cell activation (6). However, the molecular mechanism involved in LYP-CSK complex formation remains hypothetical and is still under investigation. *PTPN22* gene knockdown in mouse cells has been shown to enhance TCR signaling, resulting in hyperproliferation and activation of T cells. RA patients contain different types of antibodies directed against self-antigens. The most widely known autoantibody is Anti-Citrullinated Protein/Peptide Antibodies (ACPA/anti-CCP). ACPAs are directed against peptides and proteins that are citrullinated. During inflammation, arginine amino acid residues are enzymatically converted into citrulline residues in proteins such as vimentin, by a process called citrullination. The citrulline residues alter the structure and function of a protein, making it recognized as an antigen by the immune system (7). *PTPN22* inhibits the citrullination process by interacting with the Peptidyl Arginine Deiminase (PAD) enzyme responsible for citrullination. However, Single Nucleotide Polymorphisms (SNPs) in *PTPN22* may alter its function, which may increase the anti-CCP level (8). Thus, *PTPN22* is considered the strongest non-HLA (non-Human Leukocyte Antigen) candidate for autoimmune disease development, including RA.

SNP rs2476601 (C1858T) in the *PTPN22* gene, located in exon- 14, induces an amino acid alteration (R620W) within the proline-rich domain. This amino acid alteration disrupts the LYP-CSK interaction, leading to altered T-cell activation and proliferation. Thus, SNP rs2476601 was associated with various autoimmune diseases, including Inflammatory Bowel Disease, Type 1 diabetes, and Systemic Lupus Erythematosus (SLE) (9). Moreover, individuals with C1858T polymorphism and positive anti-CCP antibodies are highly susceptible to RA. This combination disturbs the immune tolerance, which influences anti-CCP antibody production, ultimately leading to inflammation in the joints (10, 11). Similarly, SNP rs2488457 (G1123C), located in the regulatory sequence (promoter region) of *PTPN22* gene, affects its expression by downregulating its transcription. This can lead to excessive TCR signaling, resulting in increased T cell proliferation and activation (12). On the other hand, SNP rs33996649 (G788A), a rare missense variant within exon-10 of *PTPN22* gene, induces an amino acid substitution (R263Q) in the catalytic domain of LYP. This causes conformational change in the active site of LYP and reduces its phosphatase activity, contributing toward autoimmune disease. Interestingly, SNP rs33996649 was reported as a protective allele against the development of SLE in European and American populations (8). The frequency and effect size of the *PTPN22* polymorphism vary significantly across different ethnic and geographical populations. While most studies have focused on populations of European descent, there is a paucity of data from the population we have studied. Our research provides crucial data on the prevalence and clinical significance of *PTPN22* polymorphism in the Indian cohort, addressing a key geographical gap. Based on the above-mentioned research background, this study examines the intricate relationship between specific gene loci of *PTPN22* (rs2476601, rs2488457, and rs33996649) and the predisposition to RA within the Indian ethnicities.

## 2 Materials and methods

### 2.1 Participants and sample collection

The Institutional Ethical Committee of Sri Narayani Hospital and Research Centre, Vellore, Tamil Nadu, India, authorized the study (IEC/IRB No. 29/08/07/2022), and the Declaration of Helsinki’s ethical guidelines were adhered to. All participants provided their informed consent before proceeding to the study. After obtaining ethical authorization, blood samples were collected from 239 healthy volunteers (71 male and 168 female) and 226 RA patients (49 male and 177 female), aged between 30 and 60 years. To confirm whether the sample size was sufficient to identify the significance in moderate genetic groups, the *post hoc* power analysis was carried out using the G* power v3.1.9.7 software. With a total sample size of 465, this analysis employed the chi-square (*χ*^2^) test for two independent proportions (*α* = 0.05, two-tailed). With 0.9906 (99%) power (*α* = 0.05 *χ*^2^ = 3.8414, two-tailed), the obtained sample size was adequate to perform a genetic effect study. The American College of Rheumatology/European League Against Rheumatism criteria were followed for the sample collection, and patients with other coexisting conditions were excluded.

### 2.2 Isolation of serum sample, nucleic acid, and cDNA synthesis

Serum was separated from the blood samples by centrifuging the clot after it had been allowed to coagulate without interruption. To avoid deterioration, the serum was immediately transferred to a sterile tube and stored at −20 °C. Genomic DNA was isolated from the blood (4–5 mL) mixed with an anticoagulant (Ethylenediaminetetraacetic acid) using Miller’s salting out method (13). From another 5 mL of blood, Peripheral Blood Mononuclear Cells (PBMCs) were separated using density gradient centrifugation. From PBMCs, total mRNA was extracted using an RNAiso Plus kit (Takara), and the extracted mRNA was converted into cDNA using the RT reagent kit (Takara). The thermal cycle conditions applied for cDNA synthesis were as follows: primer annealing and cDNA synthesis at 37 °C for 15 min, enzyme deactivation at 85 °C for 0.05 s, and an infinite hold at 4 °C. The quantity and quality of isolated DNA, RNA, and synthesized cDNA were determined by a Nanodrop spectrophotometer.

### 2.3 *PTPN22* gene amplification

The target region of SNPs rs2476601 (C > T), rs2488457 (G > C), and rs33996649 (G > A) from the *PTPN22* gene was amplified by Polymerase Chain Reaction (PCR). Gene sequence (Accession number (AN): NG 011432) obtained from the NCBI database was used to design the primers. The primers adapted to amplify the SNPs target regions are as follows: rs2476601 (380 bp amplification): forward- *5′CCAGCCCTACTTTTGAGCTT3′*; reverse- *5′TGCCCATCCCACACTTTATT3′*; rs2488457 (351 bp amplification): forward- *5′ GGAAGTCCTAACAACACATTTCCC3′*; reverse-*5′TCAACCACCTTGCTGACAAC3′*; rs33996649 (384 bp amplification): forward- *5′ACCCCATGTTAGAAGAGCAGA 3′*; reverse- *5′GAATTTGACCCAGGACAAGGG3′*. The reaction mixture was prepared for 30 μL, which included 6 ng of the extracted DNA as the template, 5X assay buffer, 5 pmol of both forward and reverse primers, and 1unit of HS Prime Star Taq polymerase from Takara. In Supplementary Table 1, the thermal cycle conditions applied to the reactions are listed. The resultant amplicons are processed in agarose gel electrophoresis, and the amplicon sizes were confirmed by the Gel Documentation system (14).

### 2.4 Genotyping and sanger sequencing

The High-Resolution Melting Analysis (HRMA) method is applied for exploratory purposes, such as mutation scanning and genotyping of multiple SNPs. It produces DNA melt curve profiles that are precise and sensitive enough to identify variations in nucleic acids (14, 15). The Qiagen Eva Green master mix was used to perform HRMA, incorporating the diluted (2:10) amplicons obtained from the *PTPN22* gene amplification as the DNA template. The internal primers adapted to genotype SNPs are as follows: for rs2476601, forward- *5′GGATAGCAACTGCTCCAAGGAT3′*; reverse- *5′GAACTGTACTCACCAGCTTCCT 3′*; for rs2488457, forward- *5′AGGCACTTGGGTAGACTTGT3′*; reverse- *5′GGCATTTTGGCCCTGAAAGG3′*; for rs33996649, forward- *5′CCTGAGAACTTCAGTGTTTTCAGTT 3′*; reverse- *5′ CCATTCACTATAGTTCATACCTGCG 3′*. The Bio-Rad qPCR instrument was used to generate the melt curve, and the reaction conditions for all three reactions are listed in Supplementary Table 1. To establish the reference curve, positive and negative controls were subsequently analyzed in RT-PCR, and all samples were duplicated to ensure accuracy. The Bio-Rad Precision Melt Analysis program was used to analyze the observed melt curve. To confirm the existence of SNPs in the loci, samples that revealed changes in the melt curves were further analysed using conventional Sanger sequencing (Eurofins Scientific) (15, 16).

### 2.5 Anti-CCP assay

The anti-CCP autoantibody levels in RA serum samples with SNP rs2476601 were quantified using a commercial kit (Cusabio®). Samples with anti-CCP antibody levels of 20 EU/mL was considered positive. Since SNP rs2476601 influences the level of anti-CCP antibody, the genotype frequency of rs2476601 for +ve and -ve anti-CCP was calculated to know its contribution toward RA pathogenesis.

### 2.6 LYP structural analysis by MDS

To explore the impact of SNP rs2476601 on PTPN22 structure, the wild-type PTPN22 protein structure was retrieved from UniProt (Accession: Q9Y2R2) (17) and modeled with the variant PTPN22 (R620W) using MODELLER V10 (18). Furthermore, Molecular Dynamics Simulation (MDS) analysis of the wild-type and variant proteins was carried out using GROMACS 2022 and the CHARMM36 force field (19). The proteins were simulated in a cubic box using the TIP3P water model. The wild and variant protein systems were initially energy-minimized for 1,000 steps using the steepest descent method. MDS was performed in two stages. The first stage involved an ensemble of volume, temperature, and a constant number of particles stimulation for 1 ns. The next stage involved an ensemble of a constant number of particles, pressure, and temperature for 1 ns, both conducted at 300 K. Once equilibrated in terms of pressure and density, the protein systems underwent 500 ns production MD. A Berendsen thermostat was used to sustain a constant temperature of 300 K for each simulation. For long-range Coulombic interactions, the Ewald approach was applied in conjunction with a particle mesh. A time step of 2 fs was allowed with the SHAKE algorithm, which was implemented to regulate bond lengths using hydrogen. At 1.0 nm, Coulombic and van der Waals interactions were terminated. The list of non-bonded pairs was updated every 10 steps, and for every 0.5 ps, conformations are saved. GROMACS tools were used to analyze the trajectory files and to determine the Radius of Gyration (Rg), Solvent-Accessible Surface Area (SASA), Root-Mean-Square Deviation (RMSD), and Root-Mean-Square Fluctuation (RMSF). Following that, a comparison of the variant and wild proteins was performed.

### 2.7 *PTPN22* expression analysis

*PTPN22* SNP rs2488457 (G1123C), located in the regulatory region of the gene, downregulates its expression. To correlate the link between rs2488457 and RA, the level of *PTPN22* mRNA expression was assessed in RA and control samples by the comparative threshold cycle method using qPCR. Further, the expression levels were compared between different genotypes of rs2488457 to predict the allele-specific gene expression pattern. Subsequently, mRNA expression of Glyceraldehyde-3-Phosphate Dehydrogenase (*GAPDH*) gene was used as a reference to normalize the *PTPN22* gene expression. The mRNA sequences of *PTPN22* (AN: NM_001308297.2) and *GAPDH* (AN: NP_001276674) were obtained from the NCBI database to design the primers for cDNA amplification. Primers adapted for amplification of *PTPN22* cDNA are as follows: forward, *5′-TGGATGAGGCCCAAAGCAAG-3 ′*, and reverse, *5′-TTCTTGGGCTTCTCAGCCAC-3 ′*. Similarly, for *GAPDH* cDNA amplification, the primers used are as follows: forward, *5′-ATCGTGGAAGGACTCATGAC-3 ′*; reverse, *5′-GCAGGGATGATGTTCTGGA-3′*. The reaction mixture was prepared for 10 μL, which includes 5 pmol of each primer, cDNA samples (~1,000 ng) as template, Sybr Green master mix (Takara), and RNAse-free water. The thermal cycle conditions applied for the reaction are as follows: 30 s of initial denaturation at 95 °C, 5 s of denaturation at 95 °C, 30 s of annealing at 58 °C (35 cycles), and 5-s increments of the melting curve at 65 °C to 95 °C.

### 2.8 Statistical analysis

Allele and genotype frequencies for *PTPN22* SNPs rs2476601, rs2488457, and rs33996649 were calculated. To measure their association with RA, 95% Confidence Interval (CI) of Odds Ratio (OR), Relative Risk (RR), and Chi-square (*χ*^2^) values were calculated for each genotype of rs2476601, rs2488457, and rs33996649, using MedCalc software version 23.0.2. The multiple testing corrections were calculated based on the Benjamini-Hochberg procedure, which controls the False Discovery Rate (FDR). To know the statistical differences in mRNA expressions of the *PTPN22* gene, one-way analysis of variance and Unpaired Student’s *t*-test were used. Data representation and graphs were plotted using GraphPad Software (GraphPad Prism version 10), and the data were represented as mean ± standard error of the mean. *p <* 0.05 was considered significant.

## 3 Results

### 3.1 Genotyping SNPs

**Figure 1:**
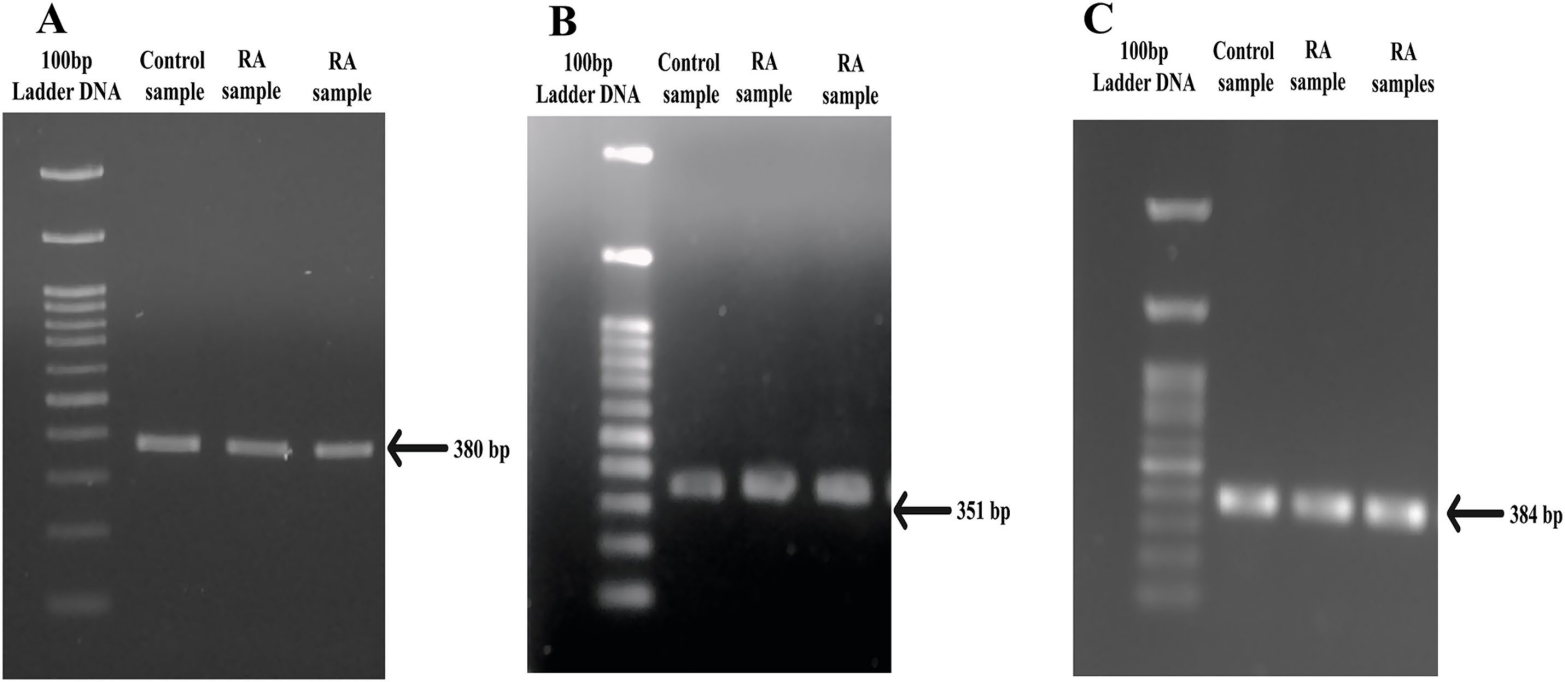
PCR amplicons of SNP target regions from *PTPN22* gene. **(A)** rs2476601 target region (380 bp), **(B)** rs2488457 target region (351 bp), and **(C)** rs33996649 target region (384 bp) of control and RA samples.

**Figure 2:**
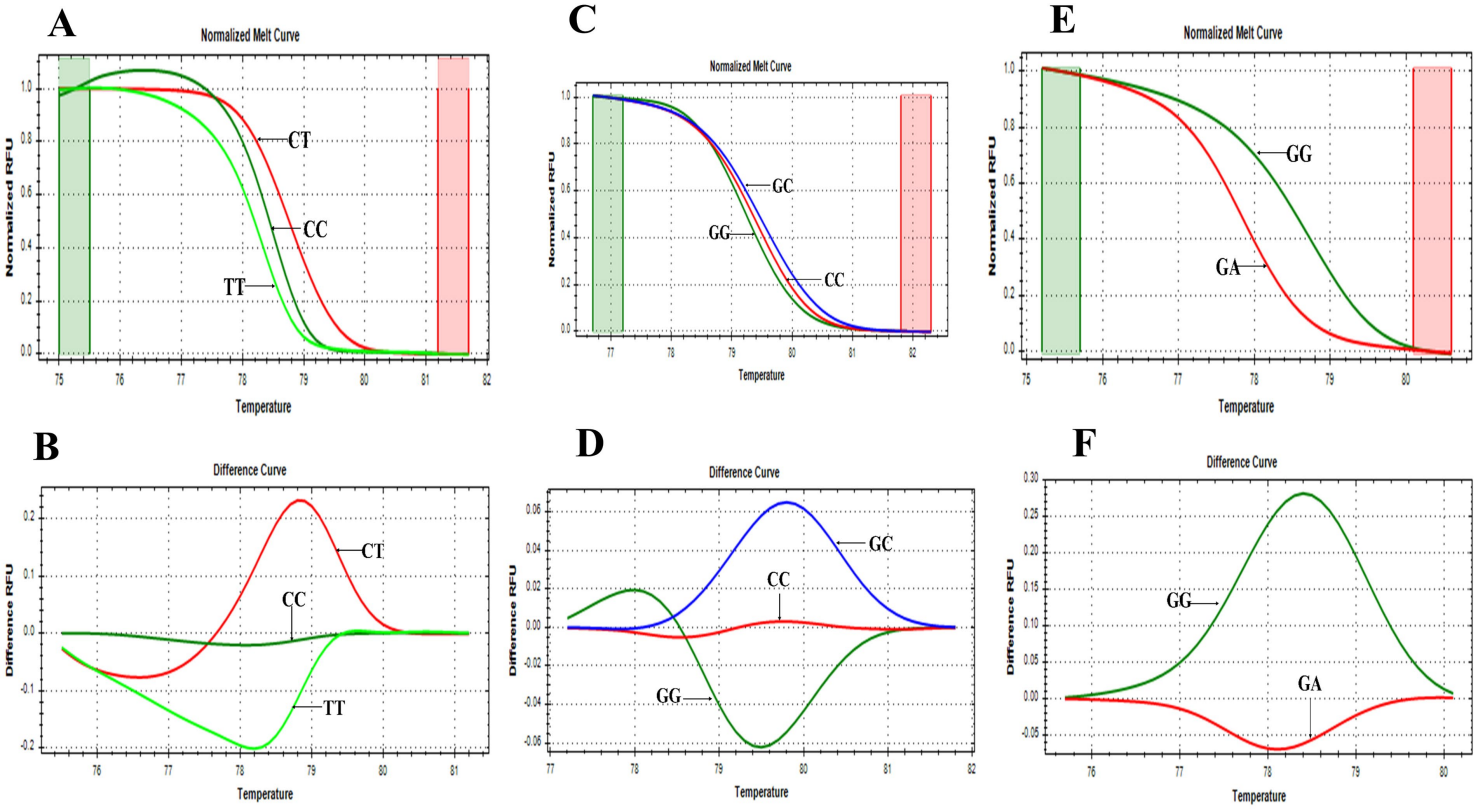
HRMA melting curves of SNPs. **(A)** Normalized melt curve of rs2476601. **(B)** Difference melt curve of rs2476601 with C/T, C/C, and T/T genotypes. **(C)** Normalized melt curve of rs2488457. **(D)** Difference melt curve of rs2488457 with G/C, G/G, and C/C genotypes. **(E)** Normalized melt curve of rs33996649. **(F)** Difference melt curve of rs33996649 with G/G and G/A genotypes.

**Figure 3:**
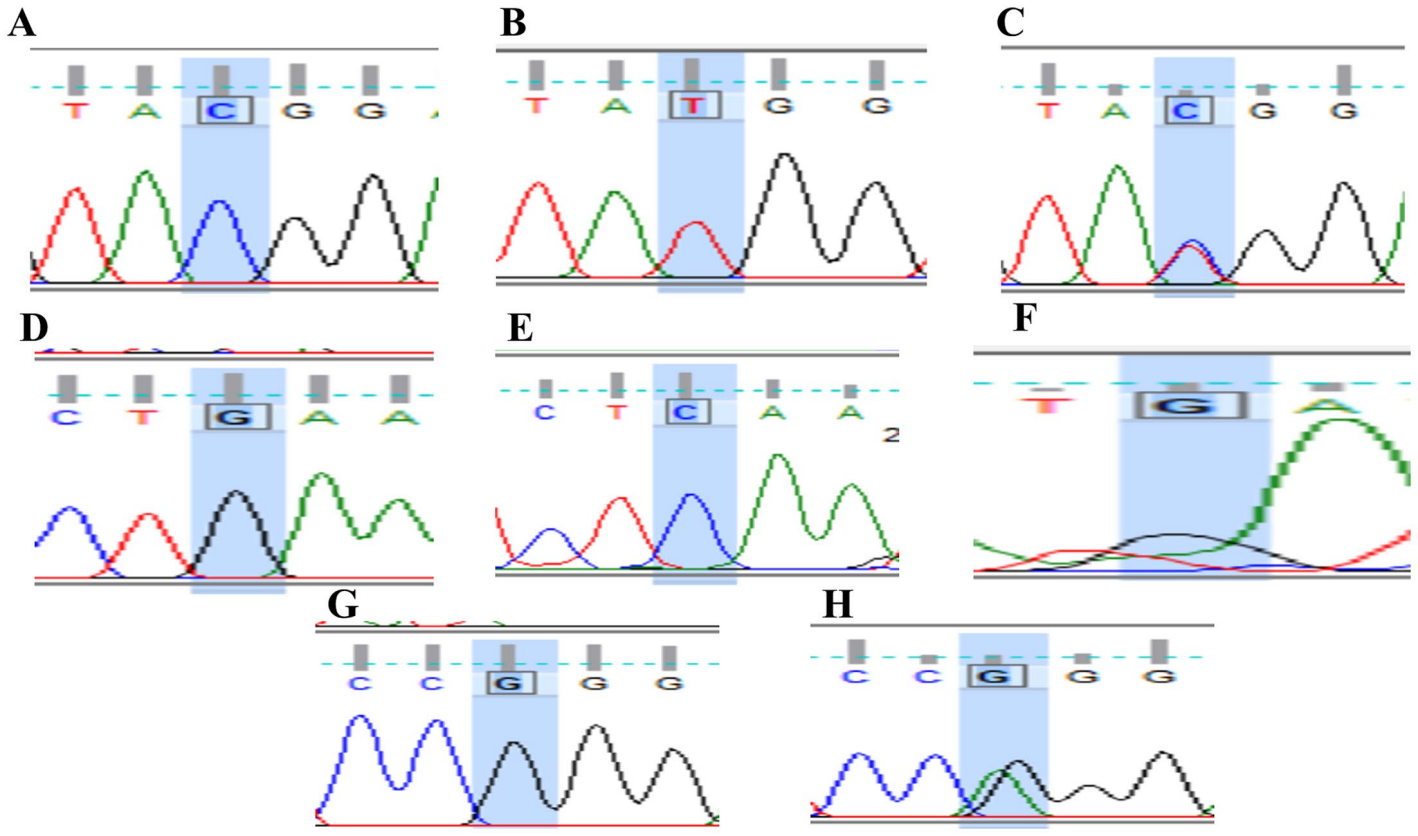
Sanger sequencing of *PTPN22* gene. SNP rs2476601 loci: **(A)** wild type - C allele, **(B)** heterozygous variant - C/T allele, **(C)** homozygous variant -T allele; SNP rs2488457 loci, **(D)** wild type - G allele, **(E)** heterozygous variant - G/C allele, **(F)** homozygous variant – C allele; SNP rs33996649 loci, **(G)** wild-type - G allele, **(H)** heterozygous variant - G/A allele.

*PTPN22* SNPs rs2476601, rs2488457, and rs33996649 were screened in both control and RA samples to predict their association with RA in the Indian ethnicity. Figures 1A–C depict the amplification of the rs2476601 (380 bp), rs2488457 (351 bp), and rs33996649 (384 bp) target sites from the *PTPN22* gene. Further, to genotype SNPs, the amplicons were analysed by HRMA. The HRMA results confirm three different clusters from normalized and difference melt curves for SNPs rs2476601 and rs2488457 (Figures 2A–D) and two different clusters for SNP rs33996649 (Figures 2E,F). To authenticate the results obtained from the HRMA, Sanger sequencing was performed on the samples exhibiting variations in the melting curves. The sequencing result of rs2476601 samples (Figures 3A–C) from each cluster confirmed the existence of C/C (wild), C/T (heterozygous variant), and T/T (homozygous variant) genotypes in rs2476601 loci on the *PTPN22* gene. Similarly, sequencing chromatograms of rs2488457 samples confirms the presence of G/G (wild), G/C (heterozygous variant), and C/C (homozygous variant) genotypes in rs2488457 loci (Figures 3D–F) and G/G (wild) and G/A (heterozygous variant) genotypes in rs33996649 loci on *PTPN22* gene (Figures 3G,H).

Table 1 represents the genotype and allele frequencies, and Table 2 represents the OR and RR of *PTPN22* SNPs rs2476601, rs2488457, and rs33996649. The dispersal of genotype frequencies of rs2476601 in control samples was C/C = 82.0%, C/T = 13.4%, and T/T = 4.6%, while in RA samples, the frequencies were C/C = 55.7%, C/T = 35.8%, and T/T = 8.4%. Compared to control samples, the C/T genotype frequency was significantly higher in RA samples. Higher OR was observed for the C/T genotype (OR = 3.6136, *p* = 0.0001, Adjusted *p*-value = 0.00045 and *χ*^2^ = 37.5228) in RA samples than control samples. The RR was also higher for the C/T genotype (RR = 2.6769, CI = 2.2789 to 5.7300) in RA samples than control samples. Therefore, in the Indian ethnicity, the *PTPN22* SNP rs2476601 C/T genotype was statistically correlated with RA etiology. The dispersal of genotype frequency of rs2488457 in controls were G/G = 44.3%, G/C = 20.5%, and C/C = 35.2%; while in RA samples G/G = 37.2%, G/C = 17.7%, and C/C = 45.1%. Higher OR (OR = 1.5179, *p* = 0.0283 Adjusted *p* value = 0.084, and *χ*^2^-4.8163) and RR (RR = 1.2339, CI = 1.0453 to 2.2042) were observed for C/C genotype in RA samples than control samples. Although a significant correlation was found between rs2488457 (C/C genotype) and RA among Indian ethnicities, subsequent analysis using an adjusted *p*-value demonstrated that this C/C genotype does not exhibit correlation. The genotype frequency of rs33996649 in control samples were G/G = 94.6%, G/A = 5.4%, and A/A = 0%; while in RA samples, G/G = 92.9%, G/A = 7.1%, and A/A = 0%. Comparing the genotypes (GG vs. GA + AA) of rs33996649, OR, RR, and CI reveals that rs33996649 has a weak association with RA. The genotype frequencies in control samples were consistent with the Hardy–Weinberg equilibrium.

**Table 1:** Genotype frequency and allele frequency of rs2476601, rs2488457, and rs33996649 SNPs in the *PTPN22* gene.

rs2476601					
Alleles	f(CC)	f(CT)	f(TT)	f(C)	f(T)
Control	0.82 (82.0%)	0.134 (13.4%)	0.0460 (4.60%)	0.887 (88.7%)	0.113 (11.3%)
RA	0.557 (55.7%)	0.359 (35.8%)	0.084 (8.4%)	0.7365 (73.65%)	0.2635 (26.35%)

rs2488457					
Alleles	f(GG)	f(GC)	f(CC)	f(G)	f(C)
Control	0.443 (44.3%)	0.205 (20.5%)	0.352 (35.2%)	0.5455 (54.55%)	0.4545 (45.45%)
RA	0.372 (37.2%)	0.177 (17.7%)	0.451 (45.1%)	0.4605 (46.05%)	0.5397 (53.95%)

rs33996649					
Alleles	f(GG)	f(GA)	f(AA)	f(G)	f(A)
Control	0.946 (94.6%)	0.054 (5.4%)	0	0.973 (97.3%)	0.027 (2.7%)
RA	0.929 (92.9%)	0.071 (7.1%)	0	0.9645 (96.45%)	0.0355 (3.55%)

**Table 2:** Odds ratio and relative risk for rs2476601, rs2488457, and rs33996649 SNPs in *PTPN22* gene.

Genotype	Odds ratio	CI	*Z*-Statistics	*p* value	*χ* ^2^	Relative risk	Adjusted *p* value	FDR
rs2476601								
CC	0.2764	0.18138 to 0.4214	5.976	0.0001	37.5228	0.6798	0.00045	0.00015
CT	3.6136	2.2789 to 5.7300	5.462	0.0001	31.7612	1.7401	0.00045	0.00015
TT	1.9025	0.8844 to 4.0928	1.9025	0.0998	2.7799	0.5688	0.2080	0.0998
rs2488457								
GG	0.7422	0.5120 to 1.0760	1.573	0.1156	2.4751	0.8380	0.2080	0.1734
GC	0.8339	0.5243 to 1.3262	0.767	0.4429	0.5884	0.8633	0.5241	0.4429
CC	1.5179	1.0453 to 2.2042	2.193	0.0283	4.8163	1.2841	0.084	0.0849
rs33996649								
GG	0.7550	0.3546 to 1.6072	0.729	0.4659	0.5333	0.9827	0.5241	0.5241375
GA	1.3245	0.6222 to 2.8197	0.727	0.4659	0.5333	1.3016	0.5241	0.5241375
AA	1.0574	0.0209 to 53.5179	0.028	0.9778	0	1.0573	0.9778	0.9778

### 3.2 Anti-CCP antibodies level in *PTPN22* (1858 T) variant

The dispersal of genotype frequencies for rs2476601 (C1858T) in RA anti-CCP + ve samples were C/C = 75.5%, C/T = 90.1%, and T/T = 84.3%, while in RA anti-CCP -ve samples, C/C = 24.6%, C/T = 9.99%, and T/T = 15.7% (Table 3). The C/T genotype frequency was significantly higher in anti-CCP + ve samples. Higher OR was observed for the C/T genotype (OR = 2.5841, *p* = 0.02) in anti-CCP + ve samples than in anti-CCP -ve samples (Table 4). This result suggests that the SNP rs2476601 increases the citrullination process and the level of anti-CCP antibodies.

**Table 3:** Genotype frequency of *PTPN22* SNP rs2476601 in RA patients, stratified by anti-CCP antibodies.

Anti-CCP	rs2476601 genotypes in anti- CCP		
	CC	CT	TT
+ve	0.754 (75.5%)	0.901 (90.1%)	0.843 (84.3%)
-ve	0.246 (24.6%)	0.099 (9.99%)	0.157 (15.7%)

**Table 4:** Multiple statistical tests of *PTPN22* SNP rs2476601 in RA patients, stratified by anti-CCP antibodies.

Parameters	CC	CT	TT
Odds ratio	0.3788	2.5841	1.2381
*p* value	0.01	0.02	0.743
CI	0.1796 to 0.7988	1.1282 to 5.9189	0.3438 to 4.4592
Adjacent *p* value	0.03	0.03	0.743
*χ* ^2^	6.7884	6.2985	0.1066
FDR	0.03	0.03	0.743

### 3.3 Impact of rs2476601 on LYP structure

**Figure 4:**
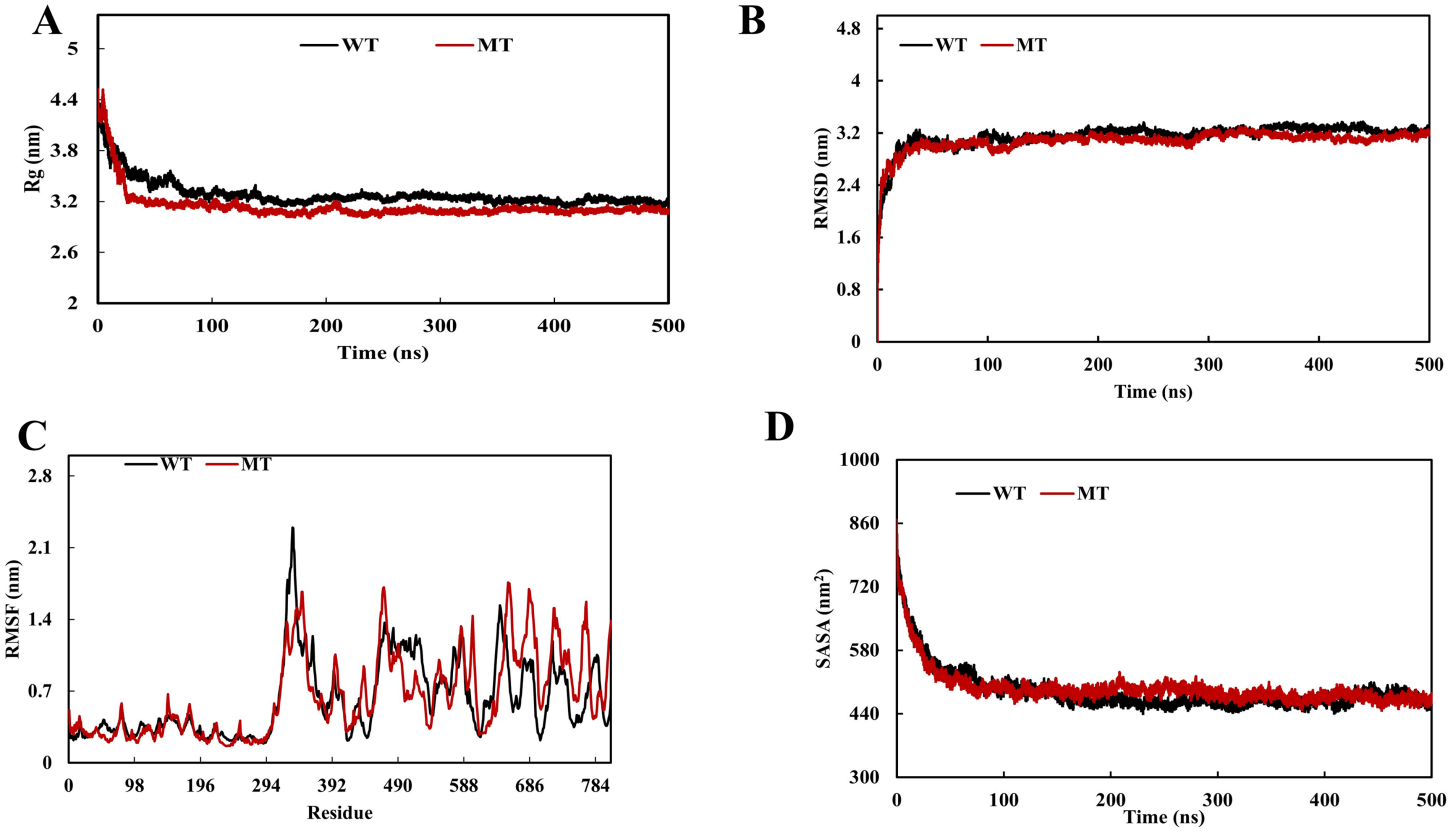
Molecular dynamics simulation (MDS) of *PTPN22* wild and *PTPN22* variant r620W protein **(A)** radius of gyration, **(B)** root mean square deviation, **(C)** root mean square fluctuation, and **(D)** solvent-accessible surface area.

To study the impact of *PTPN22* SNP rs2476601 (R620W) on LYP structure, MDS was performed. Comparison between Wild Type (WT) and variant (R620W) proteins confirmed structural and dynamic differences. The RMSD profiles (Figure 4A) revealed that both systems equilibrated in the initial ~50 ns and remained stable thereafter, with RMSD values converging around 3.0–3.2 nm. On the contrary, the variant displayed slightly increased deviations with an average RMSD value of 3.15 nm compared to WT (average RMSD value 3.08 nm), suggesting enhanced global stability. Residue-wise flexibility analysis using RMSF (Figure 4B) showed that both systems exhibited comparable fluctuations; however, the WT demonstrated increased mobility in a particular loop (329–352) with RMSFS values ranging from 1.89 to 2.29 nm, wherein variant has shown higher fluctuations at 300 (~2.0 nm), 490 (~1.5 nm), and especially at the C-terminal region 680–720 (~1.2–1.4 nm), indicating localized dynamic changes. The Rg analysis (Figure 4C) revealed that the WT system underwent a higher degree of compactness (~4.2–3.2 nm) than the variant (~4.4–3.35 nm) throughout the trajectory, consistent with increased structural stability. Similarly, SASA analysis (Figure 4D) showed increased solvent exposure in the variant compared to WT. These results indicate that the variation destabilizes the protein with a decreased degree of compactness and increased flexibility, which may influence functional interactions.

### 3.4 *PTPN22* expression level

**Figure 5:**
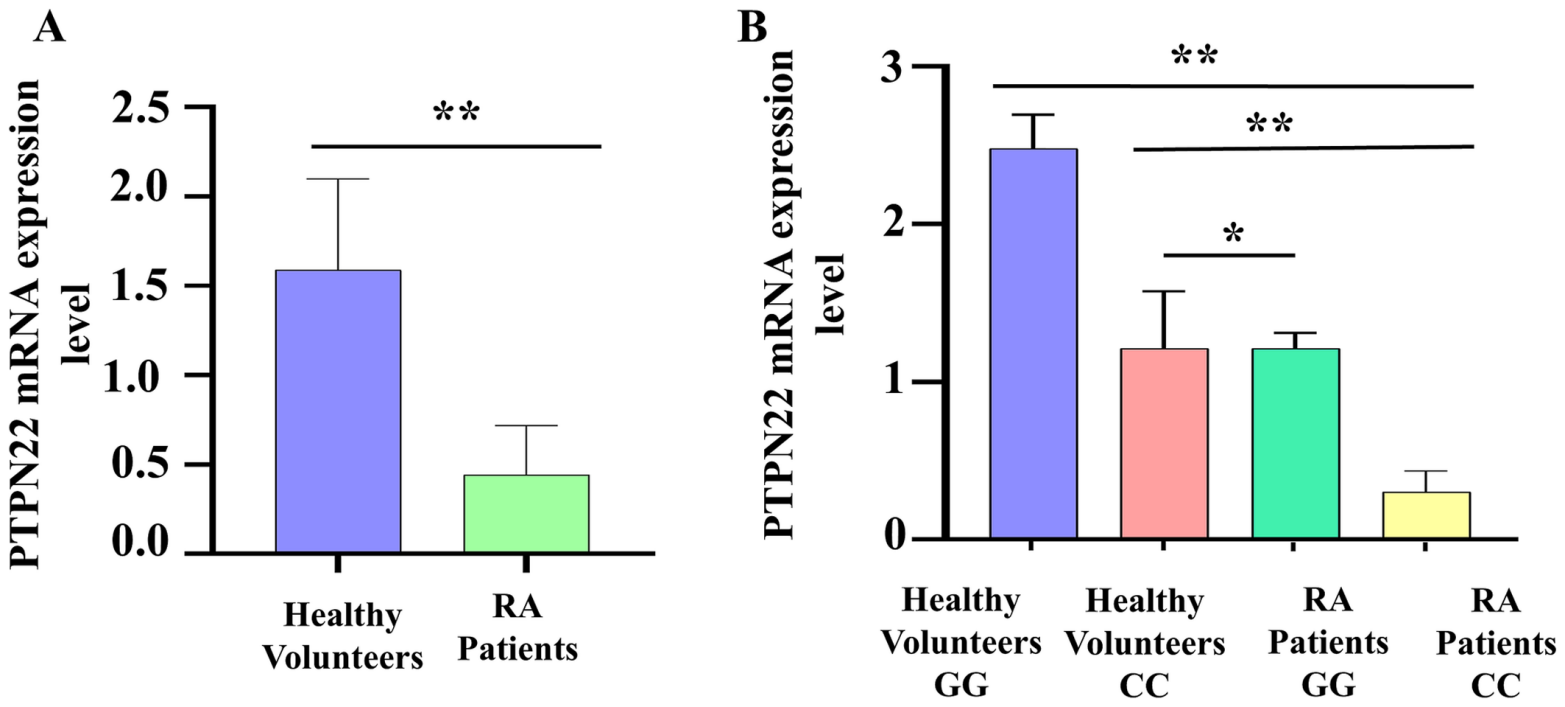
**(A)** *PTPN22* mRNA expression level between control and RA samples. **(B)** Comparison of *PTPN22* mRNA expression levels between different genotypes of SNP rs2488457.

To validate the link between SNP rs2488457 and RA pathogenesis, the mRNA expression level of *PTPN22* in RA and control samples was analysed using qPCR, and compared between the two groups as well as among different genotypes of rs2488457. The qPCR results revealed that the relative mRNA expression levels of *PTPN22* were significantly low in RA samples (0.44 ± 0.27) compared to control samples (1.58 ± 0.50, *p = 0.0350) (Figure 5A). Specifically, RA samples with C/C variant genotype showed significantly decreased expression level (0.30 ± 0.13) compared to G/G (2.47 ± 0.21, **p = 0.0001) and C/C genotypes (1.21 ± 0.36, **p = 0.0192) of control samples (Figure 5B).

## 4 Discussion

Genetic variations have different impacts on various ethnic populations in terms of diseases. Among variations, SNPs are seen in 1% of the Indian ethnicities, of which 50% are in the coding region, which includes 25% of missense and 25% of silent or synonymous. SNPs rs2476601, rs2488457, and rs33996649 located on the *PTPN22* gene were chosen for the study to determine their correlation with RA in the Indian ethnicity. LYP, synthesized from the *PTPN22* gene, interacts with the CD3 complex and dephosphorylates the TCR downstream signaling pathway kinases to inhibit the hyperactivation and proliferation of T-cells. Thus, LYP acts as a negative regulator of activation and proliferation for T-cells through the TCR signaling pathway. SNPs in *PTPN22* gene are linked to an increased risk of developing autoimmune diseases such as Alopecia Areata, RA, Graves’ Disease, Primary Sjogren Syndrome, Generalized Vitiligo, Myasthenia Gravis, Addison Disease, Systemic Sclerosis, and SLE by enhancing T-cell proliferation and activation of T-cells (20).

The *PTPN22* SNP rs2476601, located within the proline-rich domain of LYP substitutes an arginine amino acid with a tryptophan at position 620, that disrupts LYP-CSK interaction and fails to inhibit TCR signaling properly. The variant form of PTPN22 protein is less effective in dephosphorylating the kinases involved in T-cell activation. This reduced dephosphorylation leads to enhanced T-cell activation, which contributes to RA by increasing the number of effector T cells and promoting inflammation within the synovium. Our study confirms that the SNP rs2476601 has a high correlation with RA within the Indian ethnicity. The results from statistical analysis (OR, CI, and RR) indicated that the genotype C/T of rs2476601 was highly correlated with RA than the T/T and C/C genotypes. This result was supported by previous research studies in different populations. In the European population existence of rs2476601 (C/T genotype frequency - 36%) elevates the risk of RA, especially among Caucasians and Africans (21). However, in Iranian (22) and Turkish populations (23), SNP rs2476601 does not play any significant role in causing autoimmune diseases, including RA, due to its low frequency. Similar research findings were also reported in the Indian population for SLE (24). In the Northern Swedish and Western Mexican populations, RA patients carrying the C/T genotype in combination with anti-CCP antibodies had an early onset of RA (10, 11). Our study also confirms the elevated level of anti-CCP in samples with C/T genotype of rs2476601. The SNP rs2476601 can affect the interaction of PTPN22 with PAD, which potentially impacts the creation of citrullinated proteins, subsequently increasing anti-CCP antibody levels. Thus, an individual in combination with SNP rs2476601 and positive anti-CCP antibodies increases risk for developing RA. Further, to validate the impact of rs2476601 on LYP structure, MDS was performed. The results obtained from MDS reveal that the variant R620W (rs2476601) in PTPN22 adopts a less stable, more flexible conformation with altered structural integrity, which could have implications for its functional behavior and interactions. Compared to the wild type, variant R620W simulations exhibit increased fluctuations and higher RMSD values. This suggests a destabilization of the protein structure upon variant. In the catalytic domain, R620W substitution breaks the hydrophobic core and alters the protein’s folding patterns (25). The MDS on the R620W variant shows increased flexibility and altered conformational sampling of the flexible binding Widely Positioned Aspartic acid (WPD) loop and the phosphate binding loop, which plays a crucial role in substrate binding and catalytic activity. The WPD loop, which undergoes conformational changes during catalysis, exhibited a wider range of motion in variants and interruptions of loop movements, which are required for substrate recognition and dephosphorylation. The change in loop dynamics correlates with the finding that PTPN22 is involved in TCR signaling, where changes in the PTPN22 structure can have a significant impact on signaling (26).

Polymorphism within the regulatory region of the *PTPN22* gene downregulates its expression, which may lead to the loss of the gene’s ability to control TCR signaling and contribute to autoimmune conditions. To explore the contribution of SNP rs2488457 within the regulatory region of the *PTPN22* gene toward RA, the SNP was screened in RA and control samples. Additionally, to study the impact of rs2488457 on gene expression, the mRNA expression level of the *PTPN22* gene was quantified. For gene expression analysis, total RNA was isolated from the PBMCs of controls and RA samples, then reverse-transcribed into cDNA, and expression levels were analyzed using qPCR. The study confirms that the SNP rs2488457 was linked with RA within the Indian ethnic group. Statistical analysis also showed that the C/C variant genotype in SNP rs2488457 impacts RA compared to the G/C and G/G genotypes. *PTPN22* gene expression was also found to be decreased in RA samples (0.44 ± 0.27) than in the controls (1.58 ± 0.50, *p = 0.0350). When comparing genotypes, the mRNA expression in RA samples carrying the C/C genotype was significantly lower than that in the G/G wild genotype (*p* < 0.0001). SNP rs2488457 has been recently proposed as a potential cis-expression Quantitative Trait Locus in RA patients (27). This finding supports the notion that the genetic variant (SNP rs2488457, 1123C) may be crucial in downregulating *PTPN22* gene expression. However, future research should focus on elucidating the underlying molecular mechanisms for downregulating the *PTPN22* gene expression. Since *PTPN22* fails to suppress T cell activation, its reduced expression in RA patients, especially those with the C/C risk allele, will possibly contribute to the pro-inflammatory phase in RA. Our study provides compelling evidence for altered *PTPN22* mRNA expression in the Indian ethnicity, highlighting its potential role in RA pathogenesis.

Finally, we also investigated the link between *PTPN22* SNP rs33996649 and RA pathogenesis in the Indian ethnicity. The allele frequency of SNP rs33996649 in control samples was dispersed as f(G) = 97.3% (0.973), and f(A) = 2.7% (0.027), while in RA cases, it was distributed as f(G) 96.45% (0.9645) and f(A) 3.55% (0.0355). From the results, it was noted that SNP rs33996649 has a weak correlation in RA pathogenesis. The SNP rs33996649 causes an amino acid alteration at the protein’s active site, resulting in decreased phosphatase activity and reduced control over T cell activation and proliferation (9). SNP rs33996649 (788 G > A) was reported as a protective allele in SLE among the European population (28, 29). In the Chinese Uyghur population, the heterozygote genotype G/A of rs33996649, found with a 7.5% frequency, was associated with Pulmonary Tuberculosis (30). Our study confirms a strong association between the C/T genotype of SNP rs2476601 and the C/C genotype of SNP rs2488457 with RA compared to other genotypes. Conversely, SNP rs33996649 shows a weak correlation with RA development; although it might affect the function of *PTPN22*, it does not directly increase RA susceptibility. Further, larger or multicentric studies in the future are needed to assess its susceptibility in the Indian population. While *PTPN22* SNPs are not widely used for determining the efficacy of current drugs, their screening in the Indian population will offer valuable information for risk assessment, long-term monitoring, and the development of future gene-targeted therapies.

## 5 Conclusion

The results from HRMA, Sanger sequencing, and MDS suggest that the SNP rs2476601 (R620W) carrying the C/T allele in combination with anti-CCP antibodies can alter the PTPN22 protein structure and influence individuals susceptible to RA. Similarly, mRNA expression analysis and genotyping of SNP rs2488457 indicated downregulation of *PTPN22* in the RA cases carrying the C/C genotype and significantly enhanced RA development in the Indian ethnicity. The study concludes that the *PTPN22* loci rs2476601 and rs2488457 were highly correlated with RA, whereas SNP rs33996649 has no correlation with RA among the Indian ethnicities. Thus, the *PTPN22* gene can be explored as a potential biomarker and therapeutic target for RA.

## Data Availability

The datasets analyzed during the current study are available from the corresponding author upon reasonable request.
