# Supplementary material for: Exploring the impact of SNPs rs2476601, rs2488457, and rs33996649 on PTPN22 expression, structure, and anti-CCP level in rheumatoid arthritis of the Indian population: a case-control and computational study
Source: Front Med (Lausanne). 2025 Oct 21;12:1688505. doi: 10.3389/fmed.2025.1688505 (PMC12583076; doi:10.3389/fmed.2025.1688505)
Supplement: Supplementary file 1 [file Table_1.docx]

**LIST OF SUPPLEMENTARY FILES**

**Supplementary Table 1: Thermal conditions applied for amplification of rs2476601, rs2488457, and rs33996649 SNPs target region from PTPN22 gene using PCR and HRMA**

| **S. No** | **Amplification type** | **Steps** | **Temperature** | **(mins/sec)** |
| --- | --- | --- | --- | --- |
| **rs2476601** | | | | |
| 1 | PCR | Initial denaturation  Denaturation  Annealing  Extension  Final extension | 98℃  98℃  ^35 cycles^  58℃  72℃  72℃ | 3min  10sec  10sec  45sec  5min |
| 2 | HRMA (qPCR) | Initial denaturation  Denaturation  Annealing  Melting curve | 95℃  95℃  ^45 cycles^  54℃  75℃ to 85℃ | 5mins  15sec  40sec  0.1℃ increment for 5 sec |
| **rs2488457** | | | | |
| 3 | PCR | Initial denaturation  Denaturation  Annealing  Extension  Final extension | 98℃  98℃  ^35 cycles^  58℃  72℃  72℃ | 3min  10sec  10sec  45sec  5min |
| 4 | HRMA (qPCR) | Initial denaturation  Denaturation  Annealing  Melting curve | 95℃  95℃  ^45 cycles^  54℃  75℃ to 85℃ | 5mins  15sec  40sec  0.1℃ increment for 5 sec |
| **rs33996649** | | | | |
| 5 | PCR | Initial denaturation  Denaturation  Annealing  Extension  Final extension | 98℃  98℃  ^35 cycles^  58℃  72℃  72℃ | 3min  10sec  10sec  45sec  5min |
| 6 | HRMA  (q-PCR) | Initial denaturation  Denaturation  Annealing  Melting curve | 95℃  95℃  ^45 cycles^  55℃  65℃ to 85℃ | 5mins  15sec  40sec  0.1℃ increment for 5 sec |
